# Obesity and risk of hearing loss in the middle-aged and elderly: a national cohort of Chinese adults

**DOI:** 10.1186/s12889-023-15974-4

**Published:** 2023-06-01

**Authors:** Chen Zhang, Weiwei Wang, Xiaotian Chang, Siyan Zhan, Shengfeng Wang, Lei Feng, Yongfeng Song

**Affiliations:** 1 Department of Endocrinology, Shandong Provincial Hospital Affiliated to Shandong First Medical University, 324 Jing 5 road, Huaiyin District, Jinan, 250021 China; 2 National Clinical Research Center for Mental Disorders & Beijing Key Laboratory of Mental Disorders, Beijing Anding Hospital, Capital Medical University, 5 Ankang Lane, Xicheng District, Beijing, 100088 China; 3 Department of Epidemiology and Biostatistics, School of Public Health, Peking University, 38 Xueyuan Road, Haidian District, Beijing, 100191 China; 4 Department of Counseling and Clinical Psychology, Teachers College, Columbia University, 525 W 120th Street, New York, NY 10027 USA; 5 Research Center of Clinical Epidemiology, Peking University Third Hospital, 49 Huayuan North Road, Haidian District, Beijing, 100191 China; 6 Center for Intelligent Public Health, Institute for Artificial Intelligence, Peking University, 38 Xueyuan Road, Haidian District, Beijing, 100191 China; 7 Department of Endocrinology, Shandong Provincial Hospital, Shandong University, 324 Jing 5 road, Huaiyin District, Jinan, 250021 China; 8 Shandong Institute of Endocrine and Metabolic Diseases, 324 Jing 5 road, Huaiyin District, Jinan, 250021 China; 9 Central Hospital Affiliated to Shandong First Medical University, 105 Jiefang road, Lixia District, Jinan, 250013 China

**Keywords:** Waist circumference, Obesity, Overweight, Hearing loss, Cohort

## Abstract

**Background:**

The relationship between obesity and hearing loss among the middle-aged and older population remained unclear. Moreover, few studies have focused on the impact of gender on this association.

**Methods:**

This cohort study extracted the data from the China Health and Retirement Longitudinal Study, a national survey of adults aged 45 years or over. Waist circumference was categorized into three groups: normal, pre-central obesity, and central obesity. We classified BMI into four categories: underweight, normal weight, overweight, and obese. The primary endpoint was the incidence of self-reported hearing loss.

**Results:**

Of the 14,237 participants, 1972 incidents of hearing loss were identified during a median 6.9 years of follow-up. The cumulative incidence of hearing loss was 13.9% (95% CI 13.3% -14.4%). Our study showed that central obesity was significantly associated with hearing loss (HR 0.84, 95%CI 0.75–0.94), and this relationship was more prominent in males (HR 0.76, 95%CI 0.63–0.91). Among male participants, the underweight group was at the highest risk of hearing loss (HR 1.39, 95%CI 1.08–1.79). Compared with the normal weight group, the adjusted HR for hearing loss in the obese groups was 0.69 (95%CI 0.51–0.94) among men. Among female participants, only the overweight group had a lower risk of hearing loss than the normal weight group (HR 0.83, 95%CI 0.71–0.96).

**Conclusions:**

Being overweight and obese were significantly associated with a decreased risk of hearing loss, whereas being underweight was associated with an increased risk of hearing loss.

## Background

With the changes in modern lifestyles, being overweight and obese are becoming a global concern. According to the most recent national survey data, over half of the adults in China are either overweight (34.3%) or obese (16.4%) [1]. Thus, obesity and its comorbidities including hypertension, type-II diabetes mellitus, and dyslipidemia have emerged as a global epidemic [2].

Hearing loss is a highly prevalent disabling chronic condition, with more than 466 million people worldwide affected by this condition [3]. Emerging evidence indicates that obesity may affect the auditory system, a highly vascular and sensitive organ [4], through the impact of obesity-related chronic inflammation, increased oxidative stress, and hypoxia [5, 6]. However, current epidemiological studies provide mixed results regarding the associations of obesity with hearing loss. While several studies suggested that higher BMI and larger waist circumference were associated with an increased risk of hearing loss [7–9], other studies failed to support this relationship [10, 11]. Perplexingly, some studies found a positive correlation between being underweight and hearing loss [11, 12].

Recently, accumulating evidence has been supporting the “obesity paradox,” or the possible health benefits of being overweight. A survey by JAMA reports that diabetes patients who are overweight or obese had a lower mortality rate [13]. Similar results were also demonstrated in those with chronic heart failure and CAD patients with mild renal insufficiency [14, 15]. According to hospital data in Korea, cerebral infarction patients with higher BMI had a better prognosis [16]. Of note, however, it remains unclear whether there is a paradox pertaining to the relationship between obesity and hearing loss, with the relevant longitudinal data limited.

In total, there currently exist gaps in the knowledge of the associations of BMI levels and waist circumference with the incidence of hearing loss. This study was therefore aimed at exploring the associations of BMI levels and waist circumference with hearing loss, over a median 6.9 years of follow-up, utilising data from a community-based longitudinal study of Chinese elderly derived from the China Health and Retirement Longitudinal Study (CHARLS) for its large sample size, containing mid-aged and elderly participants and the thorough and detailed information on the exposure and outcome.

## Methods

### Study population

Established during 2011–2012, the CHARLS cohort enrolled 17,708 adults aged over 45 years from 450 study communities across China. The baseline data collected included: sociodemographic information, lifestyle behaviors, health status, and functioning, physical measurements (from 13,978 participants), and blood samples (from 11,847 participants). Details of the design and survey methods have been described elsewhere [17]. Three follow-up resurveys were conducted in 2013, 2015, and 2018 [18].

For the present analysis, 24,805 adults from the baseline survey (n = 17,708) and refreshment samples (n = 7097) who engaged in periodic follow-up were identified for this present analysis. Refreshment samples are new sample members recruited as the study progresses to better represent younger participants [18]. Participants younger than 45 years of age (n = 1784) or with missing values of age (n = 489) were excluded. We also excluded participants who had psychiatric problems or stroke at baseline (n = 1532), as well as had missing data for waist circumference or hearing status (n = 2828). Besides these, we excluded participants with previously and follow-up diagnosed cancer (n = 553), those who had a self-reported hearing loss or disability at baseline (n = 2789), and individuals without any follow-up interview (n = 593). Finally, a total of 14, 237 participants were included in the analysis. The Institutional Review Board of Peking University (Beijing, China) approved the study (IRB00001052-11015).

### Measurement of waist circumference

Waist circumference was measured to the nearest 0.1 cm using soft measure tape at the navel level at baseline. We categorized waist circumference into three groups: normal (< 80 cm in females and < 85 cm in males), pre-central obesity (80-85 cm in females and 85-90 cm in males), and central obesity (≥ 85 cm in females and ≥ 90 cm in males) [19].

### Measurement of body mass index (BMI)

Height and weight were measured by trained investigators with the participants standing bare feet on a stadiometer (Seca™ 213) and weight scale (Omron™ HN-286), respectively. BMI was calculated as body weight in kilograms divided by the squared height in meters to measure general adiposity. We classified BMI into four categories based on Chinese criteria [20]: underweight(< 18.5 kg/m^2^), normal weight (18.5-<24.0 kg/m^2^), overweight (24.0-<28.0 kg/m^2^), and obese (≥ 28 kg/m^2^).

### Ascertainment of outcome

The primary endpoint was the incidence of self-reported hearing loss. We defined hearing loss as a response of “Poor” to the question “Would you say your hearing is excellent, very good, good, fair, or poor?”, or a response of “Yes” to the question “Do you have one of the following disabilities: hearing problem” in the follow-up interviews. Self-reported hearing loss has been verified as a valid tool previously [21–24].

### Assessment of covariates

Similar to previous studies [8, 25, 26], we considered the following covariates: age, education (no formal education / primary school/ middle school/ college and above), occupation, current *hukou* status (rural / urban), household expenditure, smoking (never, former or current), alcohol consumption (never, former or current), diabetes (yes or no), and hypertension (yes or no). Diabetes was determined by meeting any of the following 4 criteria: (1) ≧ 126 mg/dL fasting glucose; (2) ≧ 200 mg/dL non-fasting glucose; (3) a self-reported physician diagnosis of diabetes; (4) treatment with hypoglycaemic medication [27]. Hypertension was defined as a self-reported physician diagnosis of hypertension, measured systolic blood pressure ≥ 140 mmHg, diastolic blood pressure ≥ 90 mmHg, or use of antihypertensive medications [28].

### Statistical analysis

Characteristics of the study population are described as means (SDs) for continuous variables and percentages for categorical variables, with differences evaluated using Student’s t-tests and Pearson χ^2^ tests between sexes, respectively. Person-years at risk were calculated from the baseline date until a report of hearing loss, death, loss to follow-up, or the end of follow-up(the date of wave 4 survey). The incidences of hearing loss were calculated by dividing the number of events by person-time at risk. A cox proportional hazards regression analysis was performed with the presence of hearing loss as a dependent variable and waist circumference/BMI as an independent variable to estimate the hazard ratio (HR) and 95% confidence interval (CI). Considering the potentially increasing mortality rate with age, competing risk regression models (death as competing risk) using the Fine and Gray model were performed to estimate subdistribution hazard ratios (SHRs) and 95%CIs [29]. Multivariate Cox regression and competing risk models were adjusted for age, education, occupation, current hukou status, household expenditure, smoking, alcohol consumption, diabetes, and hypertension. Additionally, we examined the joint association by classifying participants according to waist circumference and BMI because each may have independent effects on the development of hearing loss.

All analyses were performed separately for men and women using STATA statistical software, version 15.0 (StataCorp LLC). All p-values were two-tailed, and the level of statistical significance was defined as P < 0.05.

## Results

### Subjects

A total of 14, 237 individuals were included in the analysis, including 6,993 men and 7,244 women, with a mean (SD) age of 57.35 (9.19) years for men and 57.22 (9.32) years for women (P = 0.390). Compared with women, men were more likely to smoke tobacco (60.69% vs. 5.47%, P < 0.001) and consume alcohol (57.90% vs. 12.95%, P < 0.001). Female participants had a higher prevalence of diabetes (11.24% vs. 9.75%, P = 0.028), a higher proportion of central obesity (53.92% vs. 34.91%, P < 0.001), and a higher measure of BMI (overweight, 34.15% vs. 29.53%; obese, 14.94% vs. 10.22%, P < 0.001) (Table 1).

**Table 1:** Descriptive statistics for Eligible Individuals Stratified by Gender

Characteristics	Overall	Men	Women	*P*-value for sex comparison
	N = 14,237	n = 6993	n = 7244	
Age, mean (SD)	57.28 (9.25)	57.35 (9.19)	57.22 (9.32)	0.39
Smoking, n(%)^a^				< 0.001
Never	8496 (59.68)	1778 (25.43)	6718 (92.74)	
Former	1100 (7.73)	970 (13.87)	130 (1.79)	
Current	4640 (32.59)	4244 (60.69)	396 (5.47)	
Alcohol use, n(%)				< 0.001
Never	8448 (59.34)	2298 (32.86)	6150 (84.90)	
Former	802 (5.63)	646 (9.24)	156 (2.15)	
Current	4987 (35.03)	4049 (57.90)	938 (12.95)	
BMI (kg/m^2^), n(%)^b^				< 0.001
<18.5	787 (5.53)	390 (5.58)	397 (5.48)	
18.5 ~ 24.0	7066 (49.63)	3801 (54.35)	3265 (45.07)	
24.0 ~ 28.0	4539 (31.88)	2065 (29.53)	2474 (34.15)	
≥28.0	1797 (12.62)	715 (10.22)	1082 (14.94)	
Waist (cm), n(%)				< 0.001
Normal	5386 (37.83)	3355 (47.98)	2031 (28.04)	
Pre-central Obese	2504 (17.59)	1197 (17.12)	1307 (18.04)	
Central Obese	6347 (44.58)	2441 (34.91)	3906 (53.92)	
Diabetes, n(%)^c^	1496 (10.51)	682 (9.75)	814 (11.24)	0.028
Hypertension, n(%)	5170 (36.31)	2467 (35.28)	2703 (37.31)	0.012
*Hukou* ^d^				< 0.001
Urban	2721 (19.11)	1401 (20.03)	1320 (18.22)	
Rural	10,029 (70.44)	4789 (68.48)	5240 (72.34)	
Household expenditure ^e^				0.34
Bottom tertile	4419 (31.04)	2130 (30.46)	2289 (31.60)	
Middle tertile	4811 (33.79)	2380 (34.03)	2431 (33.56)	
Top tertile	4957 (34.82)	2462 (35.21)	2495 (34.44)	
Occupation^f^				< 0.001
Managers	98 (0.69%)	12 (0.17%)	86 (1.31%)	
Professionals	165 (1.16%)	59 (0.85%)	106 (1.61%)	
Technicians and associate professionals	76 (0.53%)	29 (0.42%)	47 (0.71%)	
Clerical support workers	58 (0.41%)	12 (0.17%)	46 (0.7%)	
Service and sales workers	300 (2.11%)	128 (1.84%)	172 (2.61%)	
Skilled agricultural, forestry and fishery workers	6999 (49.16%)	3609 (51.95%)	3390 (51.54%)	
Craft and related trades workers	625 (4.39%)	163 (2.35%)	462 (7.02%)	
Plant and machine operators, and assemblers	254 (1.78%)	57 (0.82%)	197 (2.99%)	
Elementary occupations	294 (2.07%)	119 (1.71%)	175 (2.66%)	
Retirement	4621 (32.46%)	2736 (39.38%)	1885 (28.66%)	
Not elsewhere classified	35 (0.25%)	23 (0.33%)	12 (0.18%)	
Hearing loss	1972 (13.85)	885 (12.66)	1087 (15.01)	< 0.001

BMI, Body mass index. There was1 missing value in smoking status ^a^, 48 missing values in BMI ^b^, 3317 missing values in diabetes ^c^, 1487 missing values in *Hukou*^d^, and 50 missing values in household expenditure ^e^, and 712 missing values in occupation ^f^

### Hearing loss

During a median follow-up of 6.9 years (74,348 person-years), 885 incident cases of hearing loss among men and 1087 cases among women were documented. The incidence rate of hearing loss per 1000 person-years for men was 24.57 (95%CI 23.00-26.25) and 28.36(95%CI 26.72–30.09) for women. The rate ratio of incident hearing loss for men compared with women was 0.87 (95%CI 0.79–0.95, P = 0.0008).

### Waist circumference and hearing loss

In multivariable-adjusted cox regression models, compared with the normal group, central obesity was significantly associated with decreased incidence of hearing loss in men (HR 0.76, 95%CI 0.63–0.91) but not in women (HR 0.89, 95%CI 0.76–1.03). A similar but not statistically significant trend was observed in the pre-central obese group (Table 2).

**Table 2:** Waist Circumference and Risk of Self-Reported Hearing Loss among CHARLS Study, HRs (95%CIs)

	Categories of Waist Circumference^‡^			
	Normal	Pre-central Obese		Central Obese
Whole cohort				
Cases	829		336	807
Deaths	364		112	275
Case/PYs(1000)	29.32 (27.39,31.39)		25.45 (22.87,28.32)	24.55 (22.91, 26.30)
Unadjusted HR (95%CI)	1.00 (Ref)		0.86(0.76,0.98) ^*^	0.83(0.76,0.92)^*^
Unadjusted SHR (95%CI) ^§^	1.00 (Ref)		0.88(0.78,1.00)	0.85(0.77,0.93)^*^
Multivariate-adjusted HR^†^ (95%CI)	1.00 (Ref)		0.93(0.81,1.07)	0.84(0.75,0.94)^*^
Multivariate-adjusted SHR^†^ (95%CI)^§^	1.00 (Ref)		0.95(0.83,1.08)	0.87(0.78,0.98)^*^
Men				
Cases	502		155	228
Deaths	266		64	137
Case/PYs(1000)	28.66(26.26,31.28)		25.13 (21.47,29.42)	18.49 (16.24,21.06)
Unadjusted HR (95%CI)	1.00 (Ref)		0.88(0.73,1.05)	0.65(0.55,0.76)^*^
Unadjusted SHR (95%CI) ^§^	1.00 (Ref)		0.89(0.75,1.07)	0.66(0.56,0.77)^*^
Multivariate-adjusted HR^†^ (95%CI)	1.00 (Ref)		1.01(0.83,1.23)	0.76(0.63,0.91)^*^
Multivariate-adjusted SHR^†^ (95%CI)^§^	1.00 (Ref)		1.04(0.86,1.26)	0.78(0.65,0.94)^*^
Women				
Cases	327		181	579
Deaths	98		48	138
Case/PYs(1000)	30.40 (27.28,33.88)		25.72 (22.24,29.76)	28.19 (25.98,30.58)
Unadjusted HR (95%CI)	1.00 (Ref)		0.84(0.70,1.01)	0.92(0.80,1.05)
Unadjusted SHR (95%CI) ^§^	1.00 (Ref)		0.85(0.71,1.02)	0.93(0.81,1.06)
Multivariate-adjusted HR^†^ (95%CI)	1.00 (Ref)		0.87(0.72, 1.06)	0.89(0.76, 1.03)
Multivariate-adjusted SHR^†^(95%CI)^§^	1.00 (Ref)		0.88(0.72,1.06)	0.91(0.79,1.06)

PYs, person-years. HR, hazards ratio. SHR, subdistribution hazard ratios. CI, confidence interval

^‡^Normal (< 80 cm in females and < 85 cm in males), pre-central obesity (80-85 cm in females and 85-90 cm in males), and central obesity (≥ 85 cm in females and ≥ 90 cm in males)

^†^ Adjusted for baseline age, education, *Hukou*, occupation, household expenditure, alcohol use, smoking status, diabetes and hypertension

^§^ Fit competing-risks regression models

^*^ P < 0.05

### BMI and hearing loss

Among men, compared with the normal weight group, the adjusted HR (95%CI) for the risk of hearing loss was 0.69 (95%CI 0.51–0.94) for the obese group, whereas underweight was inversely associated with hearing loss (HR 1.39, 95%CI 1.08–1.79). Corresponding data for women exhibited a similar trend, although the association seemed less evident than for men. The respective hazard ratios among women were 0.87 (95%CI 0.71–1.07) and 1.17 (95%CI 0.91–1.49) (Table 3).

**Table 3:** Body Mass Index and Risk of Self-Reported Hearing Loss among CHARLS Study, HRs (95%CIs)

	Categories of BMI			
	Underweight < 18.5	Normal 18.5-<24.0	Overweight 24.0-<28.0	Obese ≥ 28.0
Whole cohort				
Cases	172	1060	538	198
Deaths	98	401	175	61
Case/PYs(1000)	43.42(37.39,50.42)	28.53 (26.87,30.30)	22.69(20.85,24.69)	21.17 (18.42,24.34)
Unadjusted HR (95%CI)	1.54(1.31,1.80)^*^	1.00 (Ref)	0.79(0.71,0.87)^*^	0.74(0.63,0.86)^*^
Unadjusted SHR (95%CI) ^§^	1.45(1.24,1.70)^*^	1.00 (Ref)	0.80(0.72,0.89)^*^	0.75(0.65,0.87)^*^
Multivariate-adjusted HR^†^ (95%CI)	1.25(1.05,1.49)^*^	1.00 (Ref)	0.84(0.74,0.94)^*^	0.82(0.69,0.96)^*^
Multivariate-adjusted SHR^†^ (95%CI)^§^	1.18(0.99,1.40)	1.00 (Ref)	0.85(0.76,0.96)^*^	0.85(0.72,1.00)
Men				
Cases	84	538	205	56
Deaths	61	265	95	39
Case/PYs(1000)	44.86(36.22,55.55)	27.00(24.81,29.38)	19.46(16.97,22.31)	15.57 (11.98,20.24)
Unadjusted HR (95%CI)	1.70(1.35,2.13)^*^	1.00 (Ref)	0.72(0.61,0.84)^*^	0.58(0.44,0.76)^*^
Unadjusted SHR (95%CI) ^§^	1.57(1.24,1.97)^*^	1.00 (Ref)	0.73(0.62,0.86)^*^	0.58(0.44,0.77)^*^
Multivariate-adjusted HR^†^ (95%CI)	1.39(1.08,1.79)^*^	1.00 (Ref)	0.85(0.71,1.02)	0.69(0.51,0.94)^*^
Multivariate-adjusted SHR^†^ (95%CI)^§^	1.29(1.00,1.66)^*^	1.00 (Ref)	0.87(0.73,1.04)	0.72(0.53,0.97)^*^
Women				
Cases	88	522	333	142
Deaths	37	136	80	22
Case/PYs(1000)	42.14(34.19,51.93)	30.31(27.82,33.03)	25.26(22.69,28.13)	24.67 (20.93,29.08)
Unadjusted HR (95%CI)	1.39(1.11,1.74)^*^	1.00 (Ref)	0.82(0.72,0.94)^*^	0.81(0.67,0.97)^*^
Unadjusted SHR (95%CI) ^§^	1.33(1.07,1.66)^*^	1.00 (Ref)	0.83(0.72,0.95)^*^	0.83(0.69,0.99)^*^
Multivariate-adjusted HR^†^ (95%CI)	1.17(0.91,1.49)	1.00 (Ref)	0.83(0.71,0.96)^*^	0.87(0.71,1.07)
Multivariate-adjusted SHR^†^ (95%CI)^§^	1.11(0.88,1.40)	1.00 (Ref)	0.84(0.72,0.97)^*^	0.91(0.75,1.11)

PYs, person-years. HR, hazards ratio. SHR, subdistribution hazard ratios. CI, confidence interval. BMI, Body mass index

^†^ Adjusted for baseline age, education, *Hukou*, occupation, household expenditure, alcohol use, smoking status, diabetes and hypertension

^§^ Fit competing-risks regression models

^*^ P < 0.05

### Joint categories of waist circumference and BMI

Compared to participants with healthy waist circumference (< 80 cm for females and < 85 cm for males) and BMI (18.5–24.0 kg/m^2^), underweight male participants with healthy waist circumference had a higher risk (HR 1.43, 95%CI 1.10–1.85) of developing hearing loss, whereas overweight and obese male participants with central obesity (waist circumference ≥ 90 cm) had a lower risk for hearing loss with HRs 0.75 (95%CI 0.60–0.95) and 0.70 (95%CI 0.51–0.97), respectively. However, we did not observe a significant association among female participants (Table 4).

**Table 4:** HRs (95%CIs) for Incident Self-Reported Hearing Loss according to Crossed Classification of Waist and Body Mass Index among CHARLS Study

	Categories of Waist Circumference^‡^		
	Normal	Pre-central Obese	Central Obese
Whole cohort			
Underweight	1.26(1.04,1.52)^*^	1.17(0.58,2.36)	1.19(0.38,3.71)
Normal weight	1.00 (Ref)	1.01(0.86,1.19)	0.99(0.83,1.19)
Overweight	0.95(0.66,1.36)	0.82(0.65,1.04)	0.83(0.72,0.96)^*^
Obese	/	1.44(0.60,3.49)	0.82(0.69,0.99)
Men			
Underweight	1.43(1.10,1.85)^*^	1.37 (0.34,5.52)	/
Normal weight	1.00 (Ref)	1.09(0.85,1.38)	1.07(0.77,1.49)
Overweight	1.23(0.81,1.87)	1.01(0.75,1.36)	0.75(0.60,0.95)^*^
Obese	/	1.33(0.43,4.17)	0.70(0.51,0.97)^*^
Women			
Underweight	1.14(0.87,1.50)	1.11(0.49,2.51)	1.25(0.40,3.93)
Normal weight	1.00 (Ref)	0.95(0.76,1.20)	0.96(0.77,1.20)
Overweight	0.58(0.28,1.17)	0.62(0.42,0.90)^*^	0.85(0.71,1.03)
Obese	/	1.80(0.44,7.28)	0.86(0.68,1.08)

HR, hazards ratio. CI, confidence interval. Underweight (< 18.5 kg/m^2^), normal weight (18.5-<24.0 kg/m^2^), overweight (24.0-<28.0 kg/m^2^), and obese (≥ 28 kg/m^2^)

^‡^Normal (< 80 cm in females and < 85 cm in males), pre-central obesity (80-85 cm in females and 85-90 cm in males), and central obesity (≥ 85 cm in females and ≥ 90 cm in males)

^*^ P < 0.05

### Competing risk analysis

During the follow-up, we documented 467 deaths among men and 284 deaths among women. Overall, the strength of associations in the competing risk models slightly reduced compared with the main analysis. The association between waist circumference and hearing loss remained significant among men but not women (Table 2). With regard to BMI and hearing loss, the risk estimates attenuated in competing risk models (Table 3). Similarly, the associations between combined categories of waist circumference and BMI and hearing loss in men were not materially altered (Table 5).

**Table 5:** SHRs (95%CIs) of competing-risks regression models for Incident Self-Reported Hearing Loss according to Crossed Classification of Waist and Body Mass Index among CHARLS Study

	Categories of Waist Circumference^‡^		
	Normal	Pre-central Obese	Central Obese
Whole cohort			
Underweight	1.18(0.98,1.42)	1.12(0.56,2.23)	1.33(0.38,4.72)
Normal weight	1.00 (Ref)	1.01(0.86,1.19)	1.00(0.84,1.20)
Overweight	0.98(0.69,1.40)	0.84(0.67,1.06)	0.85(0.74,0.98)^*^
Obese	/	1.51(0.62,3.68)	0.86(0.72,1.03)
Men			
Underweight	1.32(1.02,1.72)^*^	1.30(0.31,5.35)	/
Normal weight	1.00 (Ref)	1.09(0.86,1.38)	1.09(0.79,1.51)
Overweight	1.26(0.83,1.93)	1.05(0.78,1.42)	0.77(0.61,0.97)^*^
Obese	/	1.40(0.44,4.45)	0.73(0.53,1.01)
Women			
Underweight	1.08(0.83,1.40)	1.05(0.47,2.35)	1.39(0.36,5.34)
Normal weight	1.00 (Ref)	0.95(0.76,1.18)	0.96(0.77,1.20)
Overweight	0.59(0.30,1.19)	0.62(0.43,0.89)^*^	0.87(0.72,1.04)
Obese	/	1.83(0.48,6.98)	0.90(0.72,1.12)

SHR, subdistribution hazard ratios. CI, confidence interval. Underweight (< 18.5 kg/m2), normal weight (18.5-<24.0 kg/m^2^), overweight (24.0-<28.0 kg/m^2^), and obese (≥ 28 kg/m^2^)

^‡^Normal (< 80 cm in females and < 85 cm in males), pre-central obesity (80-85 cm in females and 85-90 cm in males), and central obesity (≥ 85 cm in females and ≥ 90 cm in males)

^*^ P < 0.05

## Discussion

In this study, central obesity was significantly associated with a decreased risk of hearing loss in males but not in females over a 6.9-year period. Compared with the normal weight group, participants in the overweight and obese group had a lower risk of hearing loss, whereas those who were underweight had a higher risk of hearing loss.

In our study, being underweight was significantly associated with an increased risk of hearing loss. This pattern was especially evident for underweight participants with normal waist circumference. This finding was consistent with a recent population-based study conducted among 161,052 Korean subjects, in which the rates of hearing loss in the underweight groups were significantly higher than the normal weight group (24.9% vs. 20.4%) [12]. This relationship might be due to biological mechanisms. First, being underweight may imply decreased nutritional intake, which could result from dieting or a long-term imbalance in nutrients [30]. Decreased nutrition could aggravate the pathological degeneration of the auditory system, lead to temporal bone hypoplasia, impair resistance of hearing organs, and compromise hearing recovery [12, 31]. Second, insufficient nutritional intake, such as protein deficiency, may have a detrimental effect on the auditory system since it may disturb neurological functioning [32]. Studies of protein deficiency on rats support the view that auditory brainstem pathways were vulnerable to nutritional imbalance [33, 34]. Being underweight may be associated with more underlying comorbidities, which can lead to additional hearing loss. Noteworthy, our results are consistent with a study from Korean populations and inconsistent with other population studies. We consider that famine exposure, which both countries have suffered, might be a potential cause. Early life famine exposure and subsequent nutritional deficiencies were associated with later disease susceptibility and can increase the risk of hearing loss [31, 35, 36].The present results probably reflect an “obesity paradox” of the relationship between obesity and hearing loss. Positive effects on hearing were observed in the overweight and obese groups as well as in the central obesity group, echoing an “obesity paradox,” which has been suggested by previous studies [13–15]. Several mechanisms have been proposed. First, adipose tissue is an endocrine organ that secretes a number of adipokines [37]. Some adipokines (e.g. adiponectin, apelin, and omentin) have been shown to promote endothelial function and angiogenesis and exert anti-atherogenic effects [38–40]. Leptin is an important adipokine that regulates the mass of adipose tissue and body weight. It is produced in adipose tissue and levels increase in line with obesity. Recent research suggests that leptin may also have a beneficial role in vascular physiology [41–43]. Additionally, compared with normal-weight subjects, higher flow-mediated dilation, and lower intima-media thickness have been observed in severely obese subjects [44]. This provides further evidence for the view that severe obesity may have a protective effect against atherosclerosis. Second, a higher BMI might indicate increased tolerance to metabolic stress [45], attenuated cardiac sympathetic activity [46], and reduced neurohormonal response to stress [47]. Third, recent studies have found that obese patients had lower levels of tumor necrosis factor and other inflammatory cytokines [48]. These effects are beneficial to the auditory system, as the inner ear is highly dependent on blood supply. However, further research is needed to determine the role of multiple adipokines and to elucidate the mechanisms by which obesity is beneficial.

While our study demonstrated a possible protective role obesity had on hearing loss, previous studies find the opposite relationship. For example, several previous studies suggested that obesity was an independent risk factor for hearing loss [7–9]. A recent meta-analysis including 14 studies with 489,354 participants, also demonstrated a positive correlation between BMI and waist circumference with the risk of hearing loss [26]. One potential mechanism is that excess adiposity predisposes people to a pro-inflammatory state, leads to hypoxia and ischemic damage, increases oxidative stress, and eventually results in vascular dysfunction [4, 49]. Obesity-related vascular damage including stiffening and constriction of the internal auditory artery and reduced cochlear blood flow could exacerbate hearing loss [50]. In summary, the effect of obesity on hearing impairment remains unclear and more exploration and research are required to know for certain.

Of note, the effect of central obesity on hearing loss was solely found in males rather than in females, showing a sex-related difference in the “obesity paradox”. Similarly, previous studies reported that the obesity paradox in heart failure outcomes appears to be more pronounced in males than in females [51, 52]. Nevertheless, the gendered difference in the “obesity paradox” is not yet fully understood. Recently, some studies reported that the level of biomarkers related to inflammation and extracellular matrix remodeling was significantly lower in females than in males [53]. Thus, the anti-inflammatory effect of obesity might be more pronounced in males than in females. In addition, there exists a sex difference in fat distribution. While males tend to store more fat in the visceral depot, captured by waist circumference measurement, females tend to store fatter in peripheral subcutaneous regions [54]. In the present study, central obesity (≥ 85 cm in females and ≥ 90 cm in males) was found more strongly associated with decreased risk of hearing loss in males than females, perhaps because women have less visceral fat overall.

Strengths of this study include its nationally representative sample, large sample size, longitudinal design, validated methods to quantify BMI and waist circumference, and high participation rate. However, this study contains some limitations. First, the assessment of hearing loss was based on self-report, which may result in an underestimation of the hearing loss incidence. Nevertheless, other studies have shown that self-reported hearing loss was a valid measurement among Chinese [55] and other populations [21–24]. Second, the outcome of this study did not distinguish between etiological classifications of hearing loss. Although age-related and sensorineural hearing loss may dominate in this middle-aged and older cohort, we were unable to identify other types, such as noise-induced hearing loss and conductive hearing loss. Third, the association between obesity and incident hearing loss cannot be taken as causal because of the observational nature of this study. Although our analyses were adjusted for a wide range of known potential confounders and participants were followed up for a median of over 6 years, unmeasured confounding and reverse causation could not be fully ruled out.

## Conclusion

In conclusion, our study suggested that being overweight and obese were significantly associated with a decreased risk of hearing loss in the middle-aged and elderly over a 6.9-year period. Being underweight was observed to be positively associated with hearing loss. Further work is needed to explore the underlying mechanisms. The results from our study suggested that for the middle-aged and elderly, gaining an appropriate amount of weight may reduce the risk of hearing loss and have a positive effect on hearing function.

## Data Availability

The datasets that support the findings of the current study are available from the corresponding author upon reasonable request. CHARLS data are available via the website http://charls.pku.edu.cn/pages/data/2011-charls-wave1/en.html.
